# Gastroprotective Effects of Paeonia Extract Mixture HT074 against Experimental Gastric Ulcers in Rats

**DOI:** 10.1155/2019/3546258

**Published:** 2019-02-17

**Authors:** Young-Sik Kim, Hyo Jin Park, Hocheol Kim, Jungbin Song, Donghun Lee

**Affiliations:** ^1^Department of Herbal Pharmacology, College of Korean Medicine, Kyung Hee University, Seoul 02447, Republic of Korea; ^2^Korea Institute of Science and Technology for Eastern Medicine (KISTEM), NeuMed Inc., Seoul 02440, Republic of Korea; ^3^Department of Herbal Pharmacology, College of Korean Medicine, Gachon University, 1342 Seongnamdae-ro, Sujeong-gu, Seongnam-si, Gyeonggi-do 13120, Republic of Korea

## Abstract

**Background:**

Paeonia extract mixture HT074 is a standardized multiherbal mixture comprising extracts from* Inula britannica* flowers and* Paeonia lactiflora* roots, which are used to treat digestive disorders in traditional Korean medicine. This study was focused on elucidating the underlying mechanisms of the gastroprotective effects of HT074 in different gastric ulcer models.

**Methods:**

Gastric lesions were induced in rats by an HCl/EtOH solution, water immersion-restraint stress (WIRS), and indomethacin. Gastric secretions were studied in pylorus-ligated rats, while mucus secretions were assessed by measuring alcian blue-binding capacity of mucus in the rat model of HCl/EtOH-induced gastric ulcer. Additionally, the involvement of nitric oxide (NO) and sulfhydryl compounds in HT074-mediated mucosal protection was elucidated using their inhibitors, i.e.,* N* ^*G*^-nitro- _L_-arginine methyl ester hydrochloride (L-NAME) and* N*-ethylmaleimide (NEM), respectively. Furthermore, the effects on indomethacin-induced cell death and prostaglandin E_2_ (PGE_2_) levels were assessed in AGS cells.

**Results:**

Oral administration of HT074 significantly decreased gastric lesions induced by HCl/EtOH, WIRS, and indomethacin. Furthermore, it significantly decreased the volume, acidity, and total acidity of gastric juice in pylorus-ligated rats and increased the alcian blue-stained gastric mucus in HCl/EtOH-induced gastric ulcer in rats. Pretreatment with NEM abolished the gastroprotective effects of HT074, while L-NAME did not. In AGS cells, HT074 significantly reduced indomethacin-induced cell death and increased the PGE_2_ levels.

**Conclusions:**

These findings suggest that HT074 has gastroprotective effects against various ulcerogens, including HCl/EtOH, immersion stress, and NSAIDs. These effects are attributed to the inhibition of gastric secretions and preservation of the gastric mucosal barrier by increased mucus production, which is partially mediated through endogenous sulfhydryl compounds and PGE_2_. Based on these findings, we propose that HT074 may be a promising therapeutic agent for gastritis and gastric ulcer.

## 1. Introduction

Gastric ulcer is one of the most prevalent chronic diseases of the gastrointestinal tract [1, 2]. The main causes of gastric mucosal damage are infection by* Helicobacter pylori*, the administration of steroidal and nonsteroidal anti-inflammatory drugs (NSAIDs), stress, smoking, alcohol consumption, and nutritional deficiencies [3]. Many people are exposed to these risks, so they are vulnerable toward acquiring associated diseases, such as gastritis, that may develop into gastric ulcers [4]. If gastritis and gastric ulcers are not properly treated, they may gradually worsen and develop unexpected complications, such as bleeding or perforation [5, 6]. A gastric ulcer occurs as a result of the imbalance between the aggressive factors in the gastric system, including gastric acids or pepsin, and the protective factors, such as mucus secretion, prostaglandins, sulfhydryl compounds, nitric oxide, and antioxidants [7]. Typical treatments for gastric ulcers are acid suppressant drugs, such as type-2 histamine receptor antagonists and proton pump inhibitors [2], but they have some adverse effects. Long-term use of acid suppressants can lead to gynecomastia, impotence, osteoporotic bone fracture, and deficiencies of iron and magnesium, as well as vitamin B_12_ hypergastrinemia after discontinuation [3, 8, 9]. Gastric ulcers frequently recur and the symptoms last for a long time [5]; therefore, there is a need for drugs that can be taken long-term with minimal side effects. Currently, the main therapy of gastric ulcers is to suppress acid secretion. However, because gastric acid is only one of the many ulcerogenic factors, new treatments need to also focus on other protective factors [10]. For this reason, mucosal protective agents with relatively low side effect can be a good alternative [11]. Medicinal herbs have been used as an alternative therapeutic source for the treatment of gastric ulcers [12].

We developed the Paeonia extract mixture HT074 through a combination of 2 medicinal herbs that have significant protective effects on gastric mucosa. The Paeonia extract mixture HT074 is a standardized multiherbal mixture consisting of* Inula britannica* L. flowers and* Paeonia lactiflora* Pall. roots that are widely consumed as food and traditional medicine without safety concerns [13, 14].* Inula britannica*, also known as British yellowhead or meadow fleabane, is a plant (of the Asteraceae family) that grows in Europe, North America, and Eastern Asia [15]; its flowers are used to treat respiratory or digestive disorders in traditional Korean medicine [13]. The flowers of* I. britannica *contain sesquiterpene lactones and flavonoids, including 1-*O*-acetylbritannilactone and luteolin [15] and have anti-inflammatory, hepatoprotective, and antitumor effects [16].* Paeonia lactiflora* is a perennial peony plant (of the Paeoniaceae family) that is widely distributed in Korea, China, and Russia. Its roots are used to treat headaches, abdominal pains, and menstrual disorders in traditional Korean medicine [14]. The roots of* P. lactiflora* mainly contain monoterpene glycosides and phenolic compounds, such as paeoniflorin, albiflorin, and paeonol, and have anti-inflammatory, antitumor, antioxidant, and hepatoprotective effects [17]. The roots of* P. lactiflora* and the major active compound, paeoniflorin, in them can especially protect the gastric mucosa against HCl- and ethanol-induced gastric ulcers in mice [18, 19].

The objective of this study was to evaluate the gastroprotective effects and possible mechanisms of action of the Paeonia extract mixture HT074 in experimental models of gastric ulcers induced by HCl/EtOH, water immersion-restraint stress (WIRS), and NSAIDs. To assess the possible mechanisms behind HT074's protective effects, gastric secretions were evaluated in the pyloric ligation model. The contents of gastric wall mucus and the involvement of nitric oxide (NO) and sulfhydryl compounds, which are protective factors in the gastric mucosa, were investigated using HCl/EtOH-induced gastric ulcer models. The effects of HT074 on indomethacin-induced apoptosis and prostaglandin E_2_ (PGE_2_) content were assessed in AGS cells.

## 2. Materials and Methods

### 2.1. Sample Preparation

Dried roots of* P. lactiflora* were purchased from Daewoo Medicinal Herbs Co. (Seoul, Korea), and dried flowers of* I. britannica* were purchased from Jeseong Medicinal Herbs Co. (Seoul, Korea). The plant materials were authenticated by Hocheol Kim, and the voucher specimens were deposited in the Department of Herbal Pharmacology, College of Korean Medicine, Kyung Hee University (voucher specimen number: no. 16031403 for* P. lactiflora*, no. 16032502 for* I. britannica*). The HT074 extract mixture was manufactured in MSC Co., Ltd. (Yangsan, Korea) through the following procedure. The dried plants were individually extracted twice with distilled water for 3 h at 100°C in a reflux apparatus. Amylase was added to the* P. lactiflora* extracts to facilitate filtration and was inactivated later. The extracts were filtered, concentrated, and spray-dried with dextrin (20% for* P. lactiflora* and 10% for* I. britannica*). Powdered extracts of* P. lactiflora* and* I. britannica* were blended in a ratio of 53:47. Two lots (no. 16102850 and 16051251) were used in this study.

### 2.2. High-Performance Liquid Chromatography (HPLC) Analysis

The levels of 2 marker compounds, paeoniflorin and 1-*O*-acetylbritannilactone, were quantified by HPLC. The analysis was performed on a Waters instrument (Milford, MA, USA) equipped with a Waters 1525 binary pump, a Waters 2707 autosampler, and a Waters 2998 PDA detector using a Sunfire™ C18-column (5 mm; 250 × 4.6 mm; Waters, USA). The mobile phase consisted of 0.1% phosphoric acid (A) and acetonitrile (B) which was introduced at a flow rate of 1.0 mL/min. The gradient elution for detection was performed with the following parameters: 0–15–40–45–50-55 min, 20–20–70–70–20-20% solvent B. The detection wavelengths of paeoniflorin and 1-*O*-acetylbritannilactone were 235 nm and 210 nm, respectively. The HT074 extract was standardized to contain 21.0–31.6 mg/g of paeoniflorin and 1.50–2.26 mg/g of 1-*O*-acetylbritannilactone.

### 2.3. Animals

Seven-week-old male Sprague-Dawley strain specific pathogen-free (SPF) rats (Samtako, Osan, Korea), weighing 180–210 g, were used. The animals were acclimatized to standard laboratory conditions (23±1°C, 55±5% humidity and 12 h light/dark cycle) for 7 days before the experiments. The animals were allowed free access to food and water. During housing, animals were monitored once a day to check their health status, such as body weight, food intake, and any behavior changes. No adverse events were observed. All experimental procedures described above were approved by the Kyung Hee University Institutional Animal Care and Use Committee (Ethic no. KHUASP(SE)-17-081).

### 2.4. HCl/EtOH-Induced Gastric Lesions

The experiment was conducted as described previously by Mizui and Doteuchi [20]. After 24 h fasting, 30 rats were randomly divided into 5 groups: control; omeprazole 20 mg/kg (positive control); and HT074 30, 100, and 300 mg/kg groups. Samples were orally administered using distilled water as a vehicle. Thirty minutes after sample administration, 2 mL of 60% ethanol in a 150 mM HCl solution was administered orally to induce formation of gastric lesions. After an hour of HCl/EtOH administration, animals were sacrificed by cervical dislocation and the abdomen was opened by making a midline incision. The stomach was removed from the abdomen by cutting the bottom of the pylorus portion and upper cardiac region. The removed stomach was then fixed in 2% paraformaldehyde for 15 min at room temperature. After fixing, the stomach was opened along the greater curvature and the hemorrhagic lesion area was measured using the ImageJ program (National Institutes of Health, Bethesda, MD, USA).

### 2.5. Water Immersion-Restraint Stress-Induced Gastric Lesions

The experiment was done as described by Takagi [21]. After 24 h fasting, 30 rats were randomly divided into 5 groups: control; omeprazole 20 mg/kg (positive control); and HT074 30, 100, and 300 mg/kg groups. Samples were orally administered using distilled water as a vehicle. After an hour of sample administration, rats were placed in a stainless-steel rat cage and soaked in water using a water bath (water level to the chest of rat) at 22±1°C. After 6 h, the animals were sacrificed by cervical dislocation and the ulcerative lesion area was determined as described above.

### 2.6. Indomethacin-Induced Gastric Lesions

The experiment was conducted according to the method of Ribeiro et al., with modifications [22]. The protective effect of HT074 was compared with that of the proton pump inhibitor, omeprazole. After 24 h fasting, 30 rats were randomly divided into 5 groups: control; omeprazole 20 mg/kg (positive control); and HT074 30, 100, and 300 mg/kg groups. Samples were orally administered using distilled water as vehicle. Thirty minutes after sample administration, indomethacin (a dose of 100 mg/kg body weight was prepared by dissolving in 5% NaHCO_3_ solution) was administered orally for the induction of gastric lesions. The animals were sacrificed by cervical dislocation after 6 h of indomethacin administration. The stomachs of the rats were removed and opened along the greater curvature. The hemorrhagic lesion area was measured using ImageJ (National Institutes of Health, Bethesda, MD, USA).

### 2.7. Determination of Antisecretory Activity in Pylorus-Ligated Rats

The volume, acidity, and total acidity of the gastric juice were measured using the Shay rat ulcer method [23]. After 24 h of fasting, 24 rats were randomly divided into 4 groups: control; omeprazole 20 mg/kg (positive control); and HT074 100 and 300 mg/kg groups (n=6 animals). Thirty minutes after sample administration, the rats were anesthetized with 2% isoflurane with a mixture of 70% nitrous oxide and 30% oxygen. The abdomens of rats were opened by making a small midline incision below the xiphoid process. The pylorus portion of the stomach was lifted out slightly and ligated. Care has been taken not to cause bleeding or occlude any blood vessels. Each stomach was carefully placed back in the abdomen and the wounds were sutured. Rats were sacrificed by cervical dislocation 4 h after pylorus ligation. The stomach was removed as described in HCl/EtOH-induced gastric lesion model and the gastric juice was collected. After the collected gastric juice was centrifuged at 3,000 rpm for 10 min, the volume of gastric juice was measured, and pH was measured using pH meter (Ultrabasic benchtop meters, Denver Instruments, Denver, CO, USA). Acidity was determined by titration with 0.05 N sodium hydroxide with phenolphthalein as indicator; total acidity was calculated according to the following formula.

(1) Total  AciditymEq/4hr=Vol. of gastric  juicemL×Vol. of NaOHmL×normality  of  0.05  N  NaOH

### 2.8. Determination of Gastric Wall Mucus

Gastric mucus was estimated using the alcian blue method [24]. After 24 h of fasting, 30 rats were randomly divided into 5 groups (n=6 animals): control; omeprazole 20 mg/kg (positive control); and HT074 30, 100, and 300 mg/kg groups. Thirty minutes after sample administration, 2 mL of 60% ethanol in 150 mM HCl solution was administered orally. After an hour, the stomach was opened along the great curvature and rinsed with 0.25 M sucrose. The stomachs thus collected were stained by immersion in 0.1% w/v alcian blue solution for 2 h. The dye that complexed with mucus was eluted using 15 mL of 0.5 M MgCl_2_ solution for 18 h. The optical density of the aqueous phase was read at 605 nm.

### 2.9. HCl/EtOH-Induced Gastric Lesion in* N* ^*G*^-Nitro- _*L*_-Arginine Methyl Ester Hydrochloride (L-NAME) or* N*-Ethylmaleimide (NEM)-Pretreated Rats

This method was modified as described by Matsuda et al. [25]. After 24 h of fasting, 77 rats were randomly divided into 2 groups according to whether they were to be subjected to pretreatment of L-NAME or NEM. The 2 groups were then divided into 3 subgroups (n=6-9 animals): control, omeprazole 20 mg/kg (positive control), and HT074 300 mg/kg groups. The animals were pretreated intraperitoneally with L-NAME (70 mg/kg) and NEM (10 mg/kg) for 30 min to assess the role of nitric oxide (NO) and sulfhydryl compound. After pretreatment with the inhibitor, both vehicle and sample were administered orally; 60 min later, the rats received 60% HCl/EtOH (150 mM HCl) for induction of gastric damage. Rats were sacrificed by cervical dislocation 60 min after HCl/EtOH treatment, and the ulcerative lesion area was determined as described above.

### 2.10. Gastric AGS Cell Cultures

Human epithelial gastric cell line, AGS (KCLB no. 21739) cells, was purchased from the Korean cell line bank (Seoul, Korea). The AGS cells were grown as monolayers in the Roswell Park Memorial Institute medium (Sigma-Aldrich, St Louis, USA) with 10% heat-inactivated fetal bovine serum (FBS), 100 IU/mL penicillin, and 100 *μ*g/mL streptomycin in a humidified incubator with 5% CO_2_ in air at 37°C.

### 2.11. Cell Viability

The AGS cells were seeded in 96-well plates with a density of 1 × 10^4^ cells/well. After 24 h, the cells were treated with medium containing the HT074 extract at concentrations ranging from 50 to 1600 *μ*g/mL for 24 h. The cytotoxicity of HT074 was determined using 3-(4,5-dimethyl-2-thiazolyl)-2,5-diphenyl-2 H-tetrazolium bromine (MTT). The MTT reagents were dissolved in phosphate-buffered saline (PBS) and added to each well at a final concentration of 0.5 mg/mL. The cells were then incubated for an hour. The supernatant was removed, and 100 *μ*L of 100% dimethyl sulfoxide (DMSO) was added to each well to dissolve the formazan salts formed. Absorbance was measured at 570 nm. The percentage of cell viability was calculated using the following formula.

(2) $$\begin{array}{c} cell\;\;viability\left(\;\%\;\right)=\frac{OD\;\;control-OD\;\;sample}{OD\;\;control}\times 100 \end{array}$$

### 2.12. Indomethacin-Induced Cytotoxicity in AGS Cells

The protective effect of the HT074 extract against indomethacin-induced damage in AGS cells was assessed using the MTT assay according to the method described by Graziani et al. [26] after modifications. The AGS cells were seeded in 96-well plates with a density of 1 × 10^4^ cells/well for one day. The cells were then treated with a medium containing HT074 in two concentrations: 50 and 100 *μ*g/mL. After 24 h, the medium was replaced with serum-free medium or 800 *μ*g/mL indomethacin for 3 h. The cell viability was determined as described above.

### 2.13. Determination of PGE_*2*_ Level in AGS Cells

In order to evaluate the concentration of PGE_2_, AGS cells were seeded in 24 well plates with a density of 1 × 10^5^ cells/well plates for 24 h; the medium was then replaced with serum-free medium with HT074 in three concentrations: 50, 100, and 200 *μ*g/mL, for 1 h. The control group was treated with serum-free medium. After incubation, PGE_2_ levels were determined in the medium using an enzyme-linked immunosorbent assay (ELISA) kit (Cayman, MI, USA) according to the manufacturer's protocol.

### 2.14. Statistical Analyses

Values are presented as mean ± the standard error of the mean (SEM). The effects of the different treatments were compared by one-way analysis of variance (ANOVA) with Dunnett's* post hoc* test using GraphPad Prism 5 (GraphPad Software Inc., La Jolla, CA, USA). Values with* p *< 0.05 were considered statistically significant.

## 3. Results

### 3.1. HPLC Analysis of HT074

**Figure 1:**
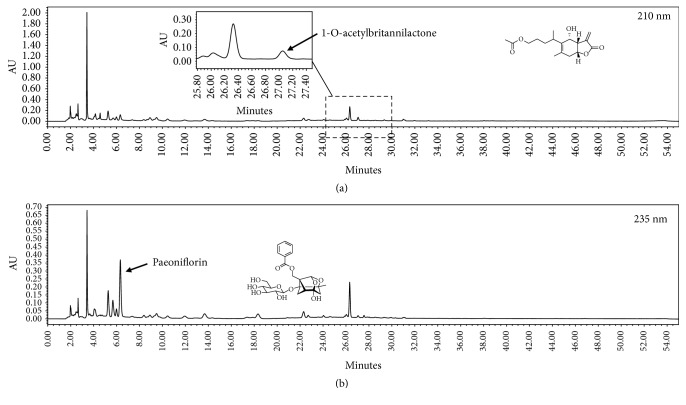
*High-performance liquid chromatograms for standardization of HT074, Paeonia extract mixture.* Arrows in (a) and (b) show the peaks of 1-*O*-acetylbritannilactone and paeoniflorin, respectively.

The representative HPLC chromatograms of HT074 are shown in Figure 1. The concentrations of paeoniflorin and 1-*O*-acetylbritannilactone were 27.55 mg/g and 1.88 mg/g, respectively, in Lot no. 16051251 and 24.93 mg/g and 1.90 mg/g in Lot no. 16102850.

### 3.2. Effect of HT074 on HCl/EtOH-Induced Gastric Lesions

**Figure 2:**
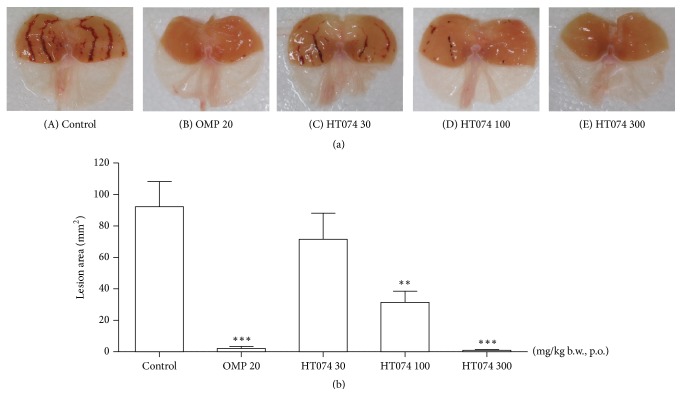
*(a) Protective effects of HT074 on HCl/EtOH-induced gastric mucosal lesions in rats.* (A–E) Representative stomach images from each group. Rats were pretreated orally with distilled water ((A), control), omeprazole 20 mg/kg (B), HT074 30 mg/kg (C), HT074 100 mg/kg (D), or HT074 300 mg/kg (E) 30 min before oral administration of HCl/EtOH.* (b) Quantification of the gastric lesion area.* Values are expressed as mean ± SEM. n=6 per group. *∗∗ p* < 0.01 and *∗∗∗ p* < 0.001 vs. control by ANOVA with Dunnett's post hoc test. OMP: omeprazole.

Administration of HCl/EtOH-induced elongated the bands of hemorrhagic lesions along the long axis of the glandular stomach (Figure 2). The mean lesion area was 92.3 ± 15.9 mm^2^ in the control group. Oral administration of HT074 at doses of 100 and 300 mg/kg significantly reduced the occurrence of HCl/EtOH-induced gastric lesions by 65.9 and 99.0%, (*p *< 0.01 and* p *< 0.001), respectively, as compared to the control group. The HT074 extract at 300 mg/kg showed gastroprotective effect that was similar to that of omeprazole; the positive control group exhibited gastric lesion reduction of 97.6%.

### 3.3. Effect of HT074 on Water Immersion-Restraint Stress-Induced Gastric Lesions

**Figure 3:**
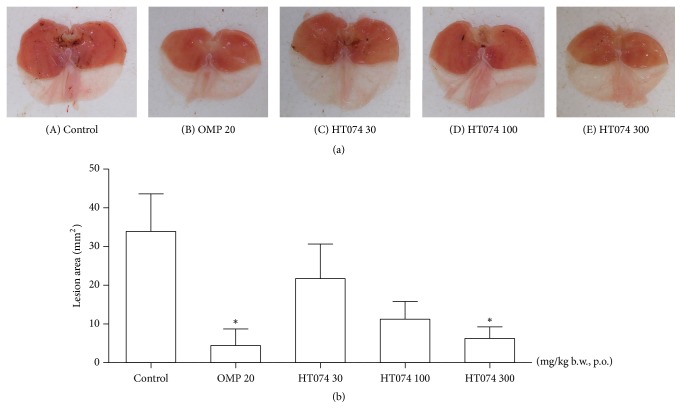
*(a) Protective effects of HT074 on the water immersion-restraint stress-induced gastric mucosal lesions in rats.* (A–E) Representative stomach images from each group. Rats were pretreated orally with distilled water ((A), control), omeprazole 20 mg/kg (B), HT074 30 mg/kg (C), HT074 100 mg/kg (D), or HT074 300 mg/kg (E) 1 h before exposure to water immersion-restraint stress.* (b) Quantification of gastric lesion area.* Values are expressed as mean ± SEM. n=6 per group. *∗ p* < 0.05 vs. control by ANOVA with Dunnett's post hoc test. OMP: omeprazole.

The WIRS caused hemorrhagic mucosal lesions in the glandular stomach (Figure 3). The mean lesion area in the control group was 33.9 ± 9.3 mm^2^. Oral administration of HT074 at a dose of 300 mg/kg and omeprazole at 20 mg/kg significantly inhibited the formation of gastric mucosal lesions by 81.5% and 87.0%, respectively, as compared to the control group (both* p *< 0.01).

### 3.4. Effect of HT074 on Indomethacin-Induced Gastric Lesions

**Figure 4:**
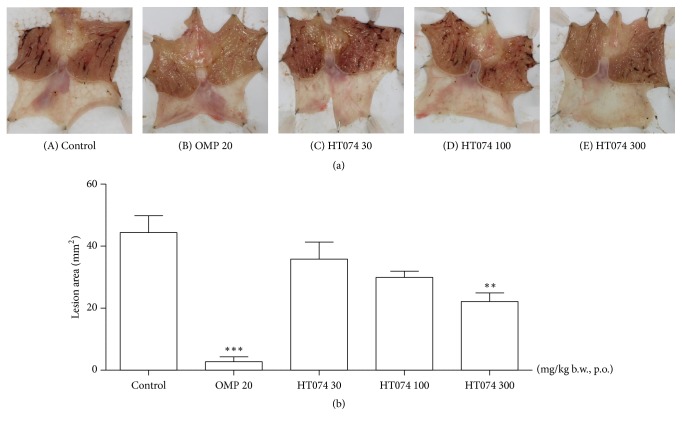
*(a) Protective effects of HT074 on indomethacin-induced gastric lesion area in rats.* (A–E) Representative stomach images from each group. Rats were pretreated orally with distilled water ((A), control), omeprazole 20 mg/kg (B), HT074 30 mg/kg (C), HT074 100 mg/kg (D), or HT074 300 mg/kg (E) 30 min before oral administration of indomethacin.* (b) Quantification of gastric lesion area.* Values are expressed as mean ± SEM. n=5-6 per group. ^*∗∗*^ *p* < 0.01, ^*∗∗∗*^ *p* < 0.001 vs. control by ANOVA with Dunnett's post hoc test. OMP: omeprazole.

Indomethacin administration caused hemorrhagic gastric lesions in the glandular stomach (Figure 4). The mean lesion area in the control group was 44.4 ± 5.41 mm^2^. Oral administration of HT074 at 300 mg/kg and omeprazole at 20 mg/kg significantly inhibited gastric mucosal lesions by 50.2% (*p *< 0.01) and 93.8% (*p *< 0.001), respectively, as compared to the control group.

### 3.5. Effect of HT074 on Gastric Secretion

The pyloric ligation caused an increase in the gastric secretions with gastric volume (7.70±0.32 mL), acidity (118.33 ± 8.50 *μ*Eq/mL), and total acidity (908.83 ± 77.74 *μ*Eq/4 h) in the control group (Table 1). In pylorus-ligated rats, oral administration of omeprazole at 20 mg/kg significantly reduced the volume (4.38 ± 0.40 mL,* p *< 0.001), acidity (30.00 ± 1.54 *μ*Eq/mL,* p *< 0.001), and total acidity (128.17 ± 8.40 *μ*Eq/4h,* p *< 0.001) of the gastric juice as compared to that in the control group. Oral administration of HT074 at 300 mg/kg significantly reduced the volume (5.37 ± 0.35 mL,* p *< 0.01), acidity (93.33 ± 5.53 *μ*Eq/mL,* p *< 0.05), and total acidity (511.17 ± 61.72 *μ*Eq/4h,* p *< 0.01) of the gastric juice as compared to that in the control group, but not at 100 mg/kg (Table 1).

### 3.6. Effect of HT074 on Gastric Wall Mucus

**Figure 5:**
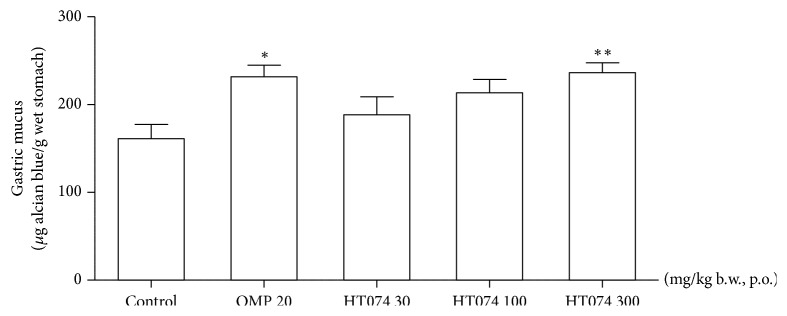
*Effects of HT074 on gastric wall mucus in HCl/EtOH-ulcerated rats.* Rats were pretreated orally with distilled water (control), omeprazole 20 mg/kg, or HT074 (30, 100, or 300 mg/kg) 30 min before oral administration of HCl/EtOH. The gastric wall mucus was quantitatively estimated using alcian blue dye binding. Values are expressed as mean ± SEM. n=6 per group. ^*∗*^ *p* < 0.05 and ^*∗∗*^ *p* < 0.01 vs. control by ANOVA with Dunnett's post hoc test. OMP: omeprazole.

In the control, the rats were subjected to gastric mucosal damage by HCl/EtOH solution; the gastric wall mucus had an alcian blue-binding capacity of 161.0 ± 16.5 *μ*g dye/g tissue (Figure 5). Oral administration of HT074 at 300 mg/kg and omeprazole at 20 mg/kg significantly increased the alcian blue-binding capacity of the gastric wall mucus to 146.7% and 143.9% in the distilled water treated group (control), respectively (*p *< 0.05 and* p *< 0.01).

### 3.7. Effect of HT074 on HCl/EtOH-Induced Gastric Lesions in L-NAME and NEM-Pretreated Rats

**Figure 6:**
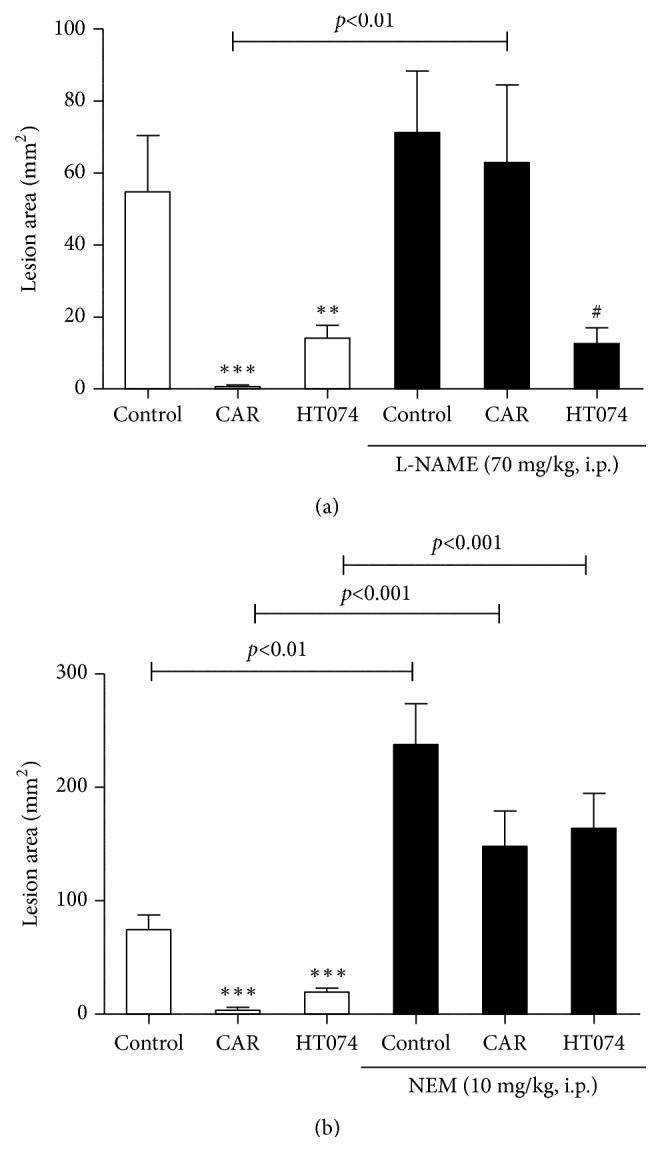
*Effects of HT074 on HCl/EtOH-induced gastric lesions in L-NAME (a) and NEM (b) pretreated rats.* Rats were pretreated intraperitoneally with saline or L-NAME or NEM and were then orally administered distilled water (control), carbenoxolone 100 mg/kg or HT074 300 mg/kg 30 min before oral administration of HCl/EtOH. Values are expressed as mean ± SEM. n=6-9 per group. ^*∗∗*^ *p* < 0.01 and ^*∗∗∗*^ *p* < 0.001 vs. control; ^#^ *p* < 0.05 vs. control + L-NAME by ANOVA with Dunnett's post hoc test. CAR: carbenoxolone.

To investigate the relevance of NO and sulfhydryl compound for the gastroprotective effect of HT074, L-NAME, and NEM, an inhibitor of NO and sulfhydryl compound, respectively, was administered before sample treatment. As shown in Figures 6(a) and 6(b), gastric lesions were produced, when the animals were orally administered HCl/EtOH or the inhibitors together with HCl/EtOH. However, in both HT074 and carbenoxolone-treated rat models without the inhibitor treatment, gastric lesions were significantly reduced. Coadministration of HT074 with L-NAME still showed significant gastroprotective effects, but the gastroprotective effect was not significant, when the animals were treated with HT074 together with NEM. Oral administration of carbenoxolone with L-NAME and NEM did not reduce gastric lesions as compared to carbenoxolone alone.

### 3.8. Effects of HT074 on Indomethacin-Induced Cell Death in AGS Cells

**Figure 7:**
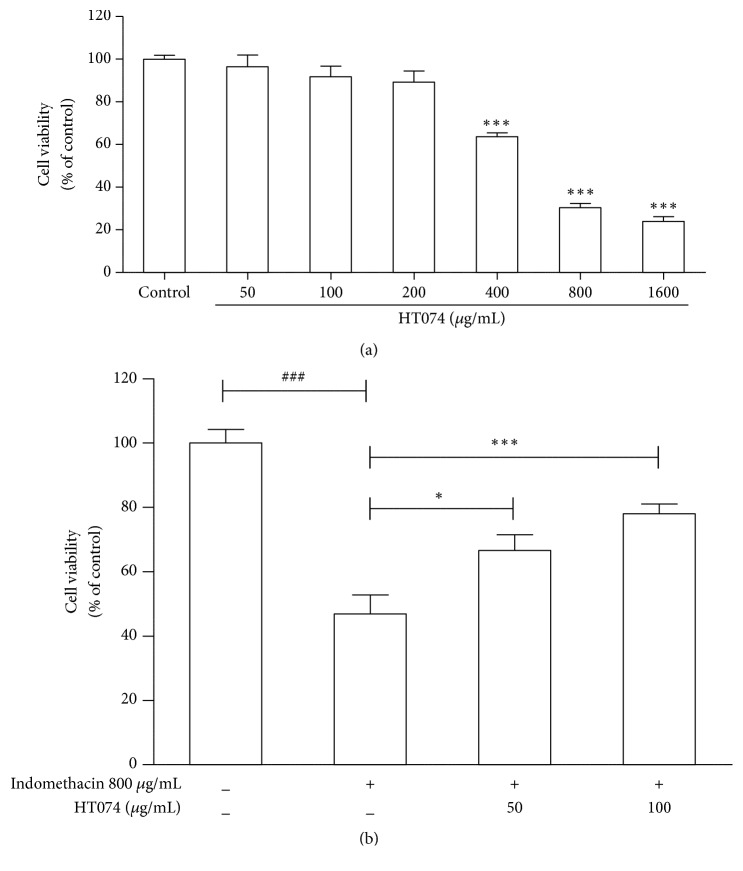
*Effects of HT074 on indomethacin-induced apoptosis in AGS cells.* (a) AGS cells were treated with HT074 for 24 h. (b) Cells were pretreated with either HT074 in serum-free medium or vehicle (serum-free medium) for 24 h and then incubated with indomethacin (800 *μ*g/mL) for 3 h. Cell viability was determined by 3-(4,5-dimethylthiazol-2-yl)-2,5-diphenyl tetrazolium bromide method. Values are expressed as mean ± SEM. ^###^ *p* <0.001, ^*∗*^ *p* < 0.05, and ^*∗∗∗*^ *p* < 0.001 by ANOVA with Dunnett's post hoc test.

To establish a range of noncytotoxic concentration for the HT074 treatment, AGS cells were treated with different concentrations of HT074 for 24 h. As shown in Figure 7(a), the concentration range of 50-200 *μ*g/mL for HT074 did not affect cell viability as compared to control. The protective effects of HT074 against indomethacin-induced apoptosis were assessed. As shown in Figure 7(b), indomethacin significantly reduced the cell viability by 46.9 ± 5.8% as compared to the untreated control cells (Figure 7,* p *< 0.001). Pretreatment with HT074 at concentrations of 50 and 100 *μ*g/mL significantly inhibited indomethacin-induced cell death with mean viability of 66.6% (*p *< 0.05) and 78.0% (*p *< 0.001), respectively.

### 3.9. Effect of HT074 on PGE_*2*_ Levels in AGS Cells

**Figure 8:**
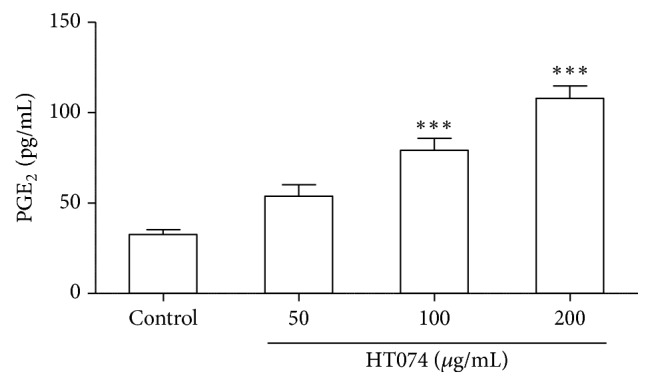
*Effects of HT074 on PGE*_*2*_ *concentration in AGS cells.* Cells were treated with either HT074 or vehicle (serum-free medium) for 1 h. Values are expressed as mean ± SEM. ^*∗∗∗*^ *p* < 0.001 by ANOVA with Dunnett's post hoc test.

As shown in Figure 8, HT074 at concentrations of 100 and 200 *μ*g/mL significantly increased the PGE_2_ levels to 79.1 (*p *< 0.001) and 107.8 pg/mL (*p* < 0.001), respectively, as compared to the control cells (32.6 pg/mL).

## 4. Discussion

Oral administration with HT074 significantly reduced gastric lesions induced by HCl/EtOH, water immersion-restraint stress, and indomethacin in rats. The HT074 extract significantly reduced acidity, total acidity, and volume of gastric juice in pylorus-ligated rats, while it increased gastric wall mucus content in HCl/EtOH-treated rats. Gastroprotective effects of HT074 were reverted by administration of NEM but not by L-NAME. The HT074 extract significantly reduced indomethacin-induced cell death and increased PGE_2_ production in AGS cells.

At doses of 100 and 300 mg/kg, HT074 significantly inhibited HCl/EtOH-induced gastric lesions by 65.9% and 99.0%, respectively, as compared to the control. The HT074 extract at 300 mg/kg had an inhibition rate as high as that of omeprazole 20 mg/kg (97.6%). Due to rapid ulcer induction and reproducibility, HCl/EtOH-induced gastric ulcer model has been used as a common and reliable model for the pathogenesis of gastritis and gastric ulcer [2, 27, 28]. Ethanol is a noxious factor that can negatively influence gastric mucosa. Ethanol can solubilize the gastric wall mucus and penetrate the gastric mucosa rapidly. Therefore, the gastric mucosa can be easily damaged by aggressive factors like HCl and pepsin [29, 30]. Furthermore, HCl/EtOH induces necrotic lesions directly by reducing defensive factors, including bicarbonate secretions and mucus production [31–35]. These results suggest that HT074 has gastroprotective effects against HCl/EtOH-induced gastric mucosal injury.

Physical and psychological stresses can play a major risk factor for the occurrence of gastric ulcerations [36–38]. The WIRS model has been reported to mimic the clinical acute gastric ulcers due to trauma, surgery, or sepsis. It has been widely used in stress-induced gastric ulcer studies [36, 38, 39]. Water-immersion and restraint stress reduces the synthesis of PGE_2_ in gastric mucosa and induces the production of reactive oxygen species [40, 41]. These changes lead to a reduction of mucosal blood flow [42–44] and mucus secretion in the gastric mucosa resulting in the development of gastric lesions [2, 45]. In this study, treatment with HT074 at 300 mg/kg significantly decreased gastric lesions that were induced by WIRS with an inhibition rate of 87%. These results suggest that HT074 has gastroprotective effects against stress-induced gastric ulcer.

Moving on, NSAIDs, that are widely used to treat pain and inflammation, are involved in gastric epithelial cells damage and promote gastric mucosal damage by decreasing endogenous prostaglandins by systemic cyclooxygenase (COX) activity inhibition [46]. Since prostaglandins are important gastroprotective factors that are involved in the decrease of acid secretion, regulation of mucosal blood flow, mucus production, and secretion [47], the use of NSAIDs causes gastric lesions in animals and humans [48]. Treatment with HT074 at 300 mg/kg significantly reduced indomethacin-induced gastric lesions by 50.2% in rats. In a previous study, indomethacin induced apoptosis in AGS cells, whereas COX-2 was necessary for their survival and proliferation [49, 50]. Particularly, it is well known that PGE_2_ protects the gastric mucosa against various aggressive factors, such as HCl/EtOH, stress, and NSAIDs [51]. The HT074 extract prevented indomethacin-induced apoptosis in the AGS human gastric epithelial cell lines, an established model for gastrointestinal toxicity by NSAIDs [52, 53]. The HT074 extract also significantly increased the PGE_2_ levels in a dose-dependent manner. Taken together, these results suggest that HT074 incurs gastroprotective effects in NSAIDs-induced gastric mucosal injury by increasing mucosal PGE_2_ levels.

The effective dose of HT074, i.e., 300 mg /kg observed in this study, corresponds to about 5.0 g of* P. lactiflora* roots and 8.2 g of* I. britannica* flowers in the daily dose of humans on being converted to human equivalent dose (HED) [54]. Since traditional medicinal practice employs 3-30 g of* P. lactiflora* roots and 3-12 g of* I. britannica* flowers for the treatment of gastrointestinal diseases [55], the effective dose used in this study falls within the conventional dose.

Gastric ulcers are caused by an imbalance between the aggressive and defensive factors of the gastric mucosa [2]. Gastric acid secretion is the main aggressive factor in gastric mucosal injury [2]. To evaluate the effect of HT074 on gastric acid secretion, we used a pyloric ligation model, which is a valid method of collecting gastric secretion [56]. Pylorus ligation increases gastric acid secretion and accumulation of intraluminal hydrochloric acid in the gastric mucosa [57–59]. As a result, the gastric mucosa itself is digested and the gastric mucosal barrier breaks down [60]. Therefore, if a substance inhibits or reduces gastric acid secretion in the pylorus ligation model, it implies that it has a gastric mucosal protective effect [12]. In the present study, pretreatment with HT074 at 300 mg/kg significantly reduced the gastric volume, acidity, and total acidity in the pylorus ligation model, but not at 100 mg/kg. Interestingly, HT074 did not significantly inhibit gastric acid secretion at a dose of 100 mg/kg but showed gastroprotective effect in the HCl/EtOH model. Therefore, it seems that the gastroprotective effect of HT074 is not completely dependent on gastric acid secretion. These results are similar to those reported for apple polyphenol extracts that were observed to have a protective effect against aspirin-induced gastric lesions without inhibition of gastric acid secretion [61]. These results suggest that HT074 partially protects the gastric mucosa by inhibiting the gastric acid secretion.

After investigating inhibition of gastric secretion by HT074, we tried to investigate the effect of HT074 on the defensive factors. Treatment with HT074 increased gastric wall mucus content in a dose-dependent manner in HCl/EtOH-induced ulceration as compared to the control. The gastric mucus is the first-line defense of the gastric mucosa against any aggressive ulcerogenic factors [62–64]. Mucus coats the entire gastric mucosa. It acts as a barrier adhering to the bicarbonate in gastric mucosa [65]. Increased mucus secretion can increase the buffering of acids in gastric juice, reduce gastric wall friction, and protect the gastric mucosa against reverse-diffusion of hydrogen ions [66]. Therefore, an increase in mucus content can inhibit the formation of gastric lesions [65]. These results suggest that an increase in the gastric mucus content is involved in gastroprotective effects of HT074 in HCl/EtOH-induced gastric mucosal injury.

To elucidate the involvement of endogenous sulfhydryl compounds and NO in the gastroprotective effect of HT074, the rats were pretreated with L-NAME or NEM before inducing gastric mucosal lesions by HCl/EtOH. It has been widely reported that sulfhydryl compounds and NO are involved in the maintenance of the gastric mucosal integrity [34, 67–69]. Sulfhydryl compounds scavenge free radicals formed during gastric injuries and, thus, help maintain the integrity of the mucosal barrier by controlling the production of mucus [70]. Endogenous NO produced by NO synthase protects the gastric mucosa from various noxious agents [71–73]. It increases gastric mucus secretion and gastric mucosal blood flow and inhibits neutrophil adherence to the endothelium [74, 75]. The gastroprotective effects of HT074 were significantly reversed by pretreatment with NEM, a sulfhydryl compound inhibitor, but not by L-NAME, an inhibitor of NO synthase in HCl/EtOH-induced gastric ulcer model. We used carbenoxolone as the reference drug in this study; it has been reported that prostaglandins, a class of SH compounds, and NO are partially involved in its mechanism of gastroprotection [76, 77]. The gastroprotective effects of carbenoxolone were significantly reversed by NEM and L-NAME. The results of this study are consistent with previous studies that reported carbenoxolone to protect the gastric mucosa by acting on NO and SH compounds [77, 78]. Taken together, these results suggest that sulfhydryl compounds are involved in the gastroprotective effects of HT074, whereas gastroprotection by HT074 is independent of NO.

It has been reported that natural products, including apple polyphenol extracts, have protective or therapeutic effects against inflammation and damage in the entire gastrointestinal system; their effects are not limited to gastric injury only [79, 80]. The HT074 extract is also expected to protect and treat various gastrointestinal diseases. Further studies on diverse gastrointestinal experimental models are needed.

## 5. Conclusions

In summary, the results of the current study revealed that HT074 exhibits a gastroprotective effect against ulcerogenic factors, including HCl/EtOH, stress, and NSAIDs. Such an effect of HT074 is attributable to the inhibition of gastric acid secretion as well as the preservation of the gastric mucosal barrier by production of the gastric mucus, which is partially mediated through endogenous sulfhydryl compounds and PGE_2_. Based on these findings, we propose that the HT074 extract may be a promising gastroprotective and therapeutic agent for treatment of gastritis and gastric ulcer.

## Figures and Tables

**Table 1:** Effects of HT074 on gastric secretion in pylorus-ligated rats.

Groups	Volume (mL)	Acidity (*μ*Eq/mL)	Total acidity (*μ*Eq/4h)
Control	7.70 ± 0.32	118.33 ± 8.50	908.83 ± 77.74
Omeprazole 20 mg/kg	4.38 ± 0.40^*∗∗∗*^	30.00 ± 1.54^*∗∗∗*^	128.17 ± 8.40^*∗∗∗*^
HT074 100 mg/kg	6.53 ± 0.45	102.50 ± 0.09	676.17 ± 66.69
HT074 300 mg/kg	5.37 ± 0.35^*∗∗*^	93.33 ± 5.53^*∗*^	511.17 ± 61.72^*∗∗*^

The volume, acidity, and total acidity of the gastric juice were measured after 4 h of pylorus ligation in rats. Values are expressed as mean ± SEM. n=6 per group. ^*∗*^ *p* < 0.05, ^*∗∗*^ *p* < 0.01, and ^*∗∗∗*^ *p* < 0.001 vs. control by ANOVA with Dunnett's *post hoc* test.

## Data Availability

The data used to support the findings of this study are available from the corresponding author upon request.

## Abbreviations

L-NAME: *N* ^*G*^-nitro- _L_-arginine methyl ester hydrochloride
NEM: *N*-ethylmaleimide
NO: Nitric oxide
HPLC: High-performance liquid chromatography
SPF: Specific pathogen-free
COX: Cyclooxygenase
ANOVA: Analysis of variance
NSAIDs: Nonsteroidal anti-inflammatory drugs
PGE_2_: Prostaglandin E_2_
PBS: Phosphate-buffered saline
FBS: Fetal bovine serum
WIRS: Water immersion-restraint stress
DMSO: Dimethyl sulfoxide
ELISA: Enzyme-linked immunosorbent assay
SEM: Standard error of the mean
HED: Human equivalent dose
MTT: 3-(4,5-dimethyl-2-thiazolyl)-2,5-diphenyl-2 H-tetrazolium bromine.
